# Childhood cerebral visual impairment subtype classification based on an extensive versus a limited test battery

**DOI:** 10.3389/fnins.2023.1266201

**Published:** 2023-10-26

**Authors:** Jannet Philip, Bianca Huurneman, Nomdo M. Jansonius, Antonius H. N. Cillessen, Frouke N. Boonstra

**Affiliations:** ^1^Royal Dutch Visio, National Foundation for the Visually Impaired and Blind, Huizen, Netherlands; ^2^Behavioural Science Institute, Radboud University, Nijmegen, Netherlands; ^3^Department of Cognitive Neuroscience, Donders Institute for Brain, Cognition and Behaviour, Radboud University Medical Centre, Nijmegen, Netherlands; ^4^Department of Ophthalmology, University Medical Center Groningen, University of Groningen, Groningen, Netherlands; ^5^Graduate School of Medical Science, University Medical Center Groningen, University of Groningen, Groningen, Netherlands

**Keywords:** cerebral visual impairment, childhood, subtyping, classification, visual function, eye movements, visual fields, optic disk

## Abstract

**Purpose:**

To classify CVI subtypes and compare the added value of an extensive test battery over a limited test battery in subtype classification of cerebral visual impairment (CVI) in children.

**Methods:**

Seventy-five children with a clinical diagnosis of CVI (median [IQR] age: 9 [7–12] years) were identified from the medical records. The extensive test battery included visual acuity, contrast sensitivity, ocular alignment, eye movement analysis, visual field analysis, optic nerve head evaluation, and evaluation of visual perception. The limited test battery included visual acuity, contrast sensitivity, ocular alignment, and evaluation of visual perception. Principal component analysis (PCA) followed by cluster analysis was done, for both test batteries separately, to determine the optimum subtype classification for CVI.

**Results:**

Fifty-one participants with an extensive test battery with mild to moderate visual impairment were included in the main analysis. This resulted in four CVI subtypes for the extensive test battery (subtle characteristics, higher-level visual function deficits, lower-level visual function deficits, and higher- and lower- level visual function deficits) and three CVI subtypes for the limited test battery (subtle characteristics, higher-level visual function deficits, and higher- and lower- level visual function deficits). There were significant differences between the subtypes for 9 out of 10 measures of the extensive and all 4 measures of the limited test battery (*p* < 0.05). The subtle characteristics subtype (extensive *n* = 19, limited *n* = 15) showed near normal lower and higher-level visual functions in both test batteries. The higher-level visual function deficits subtype (extensive *n* = 18, limited *n* = 24) showed near normal visual acuity combined with significant visual perceptual deficits in both test batteries; accompanied by visual pathways defects and abnormal eye movement behavior in the extensive test battery. The higher- and lower- level visual function deficits subtype (extensive *n* = 4, limited *n* = 12) showed both higher and lower-level visual function deficits in both test batteries, but application of the extensive test battery revealed additional visual pathways defects and abnormal eye movement behavior. The lower-level visual function deficits CVI subtype (extensive *n* = 10) was a new subtype identified by the extensive test battery. This subtype showed lower-level visual function deficits together with abnormal eye movement measures.

**Conclusion:**

This data-driven study has provided meaningful CVI subtype classifications based on the outcomes of various key functional and structural measures in CVI diagnosis. Comparison of the extensive test battery to the limited test battery revealed the added value of an extensive test battery in classifying CVI. The outcomes of this study, therefore, have provided a new direction in the area of CVI classification.

## Introduction

Cerebral visual impairment (CVI) is a visual disorder caused by damage to the retrochiasmal visual pathways of the brain in the absence of any major ocular disorder (Boot et al., 2010). With previous definitions focusing only on anatomical landmarks (Fazzi et al., 2007), a recent definition portrays CVI as “a verifiable visual dysfunction that cannot be attributed to disorders of the anterior visual pathway or any potentially co-occurring ocular impairment” (Sakki et al., 2018). In the Netherlands, the prevalence of CVI among children with low vision was reported to be 27%. CVI was the most common cause of visual impairment in children (Bosch et al., 2014). In individuals with CVI, there is a heterogeneous clinical presentation (Fazzi et al., 2007; Boot et al., 2010; Dutton, 2013; Lueck et al., 2019; Ortibus et al., 2019). Along with visual deficits, there are coexisting disabilities in a majority of children multiple impairments are seen (Bosch et al., 2014). As a result, diagnosing CVI is a challenge.

Recently, a multidisciplinary guideline for diagnosing CVI has been published (Boonstra et al., 2022). This guideline mentions a series of key features that should be assessed in a child with CVI. These features included a structured medical history and functional vision questionnaires, orthoptic and ophthalmic assessments including visual acuity, contrast sensitivity, fixation and eye movements, crowding, accommodation, visual fields, and retinal and optic nerve head evaluation, neuropsychological assessments, neuroradiological evaluation, and a genetic workup. Although the guideline consists of a step-wise, elaborate view on standardizing diagnosis, there is no information about CVI classification or subtyping provided. Classifying CVI into subtypes will aid in: (i) a better understanding of the disease, (ii) simplifying the diagnosis by reduction of diagnostic measures in multiple impaired children and (iii) framing educational and interventional strategies.

There are several classification systems for CVI in the literature and each of these vary in nature and applicability. One of these systems is practical (Philip and Dutton, 2014), one is descriptive (Sakki et al., 2018), and one is based on structural etiologies (Boot et al., 2010). A recent classification system was based on functional vision measures and is, as opposed to the other systems, primarily data-driven (Sakki et al., 2021). The authors of this classification system used a cluster analysis with visual acuity, contrast sensitivity, stereopsis, and a visual perception index as the input. They included children of 5–16 years of age with a suspicion or diagnosis of congenital CVI. The analysis resulted in three CVI subtypes: group A1 with normal or near normal outcomes for all measures, group A2 with lower outcomes for all measures compared to group A1, and group B that consisted of low functioning children who were not included in the cluster analysis (because a systematic assessment was not possible). Several of the key features listed in the multidisciplinary guidelines (Boonstra et al., 2022) were not incorporated in the classification system of Sakki et al. (2021). For example, information about the aspect of the optic disk, visual fields, and fixation and eye movements was lacking.

In CVI caused by pre- and perinatal damage, thinning of the retinal nerve fiber layer (RNFL) and retinal ganglion cell layer (RCGL; Jacobson et al., 2019), and a small optic disk or a disk with pronounced excavation (Ruberto et al., 2006) have been reported. VFDs such as inferior defects, homonymous hemianopia, and concentric defects (Bosch et al., 2014) are commonly seen in CVI. A combined evaluation of structure (RNFL, RGCL, and optic disk) and function (visual fields) helps to understand the integrity of the visual pathways in CVI (Jacobson et al., 2020). Several studies provided evidence for ocular motor dysfunction in children with CVI (Kooiker et al., 2015; Ben Itzhak et al., 2023). Measuring fixation and eye movements in children with CVI can be used to estimate visual information processing (spatial orientation, visual attention, recognition, memory etc.) also in preverbal children and children with developmental delays.

In this study, these missing key features from the multidisciplinary guidelines (aspect of the optic disk, visual fields, and fixation and eye movements) were added to the data-driven approach as initiated by Sakki et al. (2021). We performed a principal component analysis (PCA) followed by a cluster analysis on a complete retrospective dataset of CVI patients with mild to moderate visual impairment (in whom all the relevant key features were measured). For comparison, we performed the same analysis on the same dataset, but with a limited number of key features: visual acuity, contrast sensitivity, strabismus, and visual perception measures, like those used by Sakki et al. (2021). This should provide information about firstly, the CVI subtypes with each test battery and secondly, the added value of the extensive list of key features compared to a limited set, which is easier to collect in daily clinical practice.

## Materials and methods

### Participants

In total, 75 patients with a clinical diagnosis of CVI (40 boys and 35 girls) were included in the study. The medical records of children diagnosed with CVI between 2018 and 2022 were considered for a retrospective data analysis. The inclusion criteria were (i) calendar age of 1–18 years, (ii) a clinical diagnosis of CVI as confirmed by a pediatric ophthalmologist, and (iii) all CVI etiologies and inclusion irrespective of developmental age. We excluded those with coexisting ocular diseases (except for patients with eye diseases considered to be comorbid with CVI, such as optic nerve damage or retinopathy of prematurity).

For the use of data collected during regular clinical care an exemption from ethical review was obtained from the METC of Oost-Nederland (MEC 2021–13169). Informed consent was obtained from each patient’s parent or caregiver. We adhered to the tenets of the Declaration of Helsinki (2013) for research involving human subjects.

### Data collection

#### Procedure

All medical records were retrospectively reviewed by one independent researcher. This included screening and filtering of the records based on the inclusion and exclusion criteria, and data extraction. Data were collected in a single visit that was part of routine care by an expert multidisciplinary team of professionals. The pediatric history taking and functional vision assessments were carried out by trained orthoptists. The assessment of visual perception was done by neuropsychologists. Motor function tests, both gross and fine, were carried out by child-physiotherapists. Evaluation of the optic nerve head (ONH) and the diagnosing of CVI were done by a pediatric ophthalmologist. Any discrepancies or doubts regarding CVI diagnosis were discussed with the team in order to make a final decision on whether to include or exclude the patient.

#### Measurements

Two test batteries were compared in this study, an extensive and a limited test battery. The extensive test battery included assessment of visual acuity, contrast sensitivity, ocular alignment, eye movement analysis, visual field analysis, ONH evaluation, and evaluation of visual perception. The limited test battery, resembling the tests included by Sakki (2021), comprised assessment of visual acuity, contrast sensitivity, ocular alignment, and evaluation of visual perception. To see the overview of the completeness of the visual function assessments see Supplementary Table S1. The tests are described in more detail below.

##### Visual acuity

The choice of the visual acuity chart was based on the child’s developmental age. In preverbal children with a developmental age up to 2 years, Teller Acuity Cards (TAC) were used (Mash and Dobson, 1998). In children between 2 and 4 years, the Cardiff Acuity Test (Adoh and Woodhouse, 1994) or Kay pictures (Shah et al., 2012) were used. In verbal children (3–6 years) in the preschool age group, distance visual acuity was measured with the Lea symbol chart (Repka, 2002). In children above 6 years, visual acuity was recorded with a number chart or with Lea symbols (using the Vision Inspector Pro (VIP) software.^1^) Near visual acuity was measured using a Lea near symbol chart.^2^ Visual acuity was measured monocular and binocular with optimal refractive correction; the binocular value was used for analysis. Presence of refractive error was defined as follows: a spherical equivalent below −0.75 D (myopia) or + 2.00 D or above (hyperopia), or astigmatism of 1.00 D or more (Saunders et al., 2010; Wilton et al., 2021).

##### Contrast sensitivity

In preverbal children with a developmental age below 2 years, Hiding Heidi low contrast face test (Leat and Wegmann, 2004) was used for assessing contrast sensitivity. The Cardiff Contrast Sensitivity Test (Adoh and Woodhouse, 1994) was performed in children in the preschool age group. The standard distances were used. In verbal children, Lea symbol low contrast (10 M) chart (Leat and Wegmann, 2004) or the Groningen edge contrast chart (Aart Kooijman et al., 1994) at 3 m was performed. Contrast sensitivity was measured monocular and binocular with optimal correction; the binocular value was used for analysis.

##### Ocular alignment

An estimation of ocular alignment was made using a cover test for distance and near (Hull et al. 2017). The outcome was graded as normal or abnormal ocular alignment (i.e., manifest strabismus) for the classification analysis.

##### Eye movement analysis

Eye movements were recorded using a video-based 24-inch integrated infrared eye tracker system (TFT eye tracker sampling at 60 Hz; Tobii T60XL, Tobii Corporation, Danderyd, Sweden). Varying cartoons and moving blocks are presented on the screen to analyze the spontaneous visual behavior [for more information about the procedure see Kooiker et al. (2014)]. Three outcome measures were extracted for analysis: (i) reaction time fixation to cartoon stimulus (RTFc, in ms), (ii) reaction time fixation to a motion stimulus (RTFm, in ms), and (iii) gaze fixation area to the cartoon stimulus (GFA, in deg^2^).

##### Visual field testing

In children with a developmental age lower than 5 years, double arc perimetry with Stycar balls (Dobson et al., 1998) was done. In children with developmental age of 5 years or higher, kinetic perimetry was performed. Either one of two kinetic perimetry techniques was used: Octopus kinetic perimetry (Bjerre et al., 2014) or Goldmann perimetry. In children capable of providing reliable responses, static visual field testing using Octopus was carried out (Schmied, 1980). The 30G TOP strategy was used. The testing time takes between 2 and 4 min per eye. Sometimes one or more techniques were combined (static and kinetic) to assess the visual fields reliably. The outcomes were dichotomized as VFD absent or present.

##### Optic nerve head evaluation

Optic nerve head evaluation was done with slit lamp biomicroscopy with 90 D lens or by indirect ophthalmoscopy with 20 D lens. We assessed size and color. The size of the optic disk was graded as small optic disk yes or no; the color was graded as pale optic disk yes or no (Ruberto et al., 2006).

##### Visual perception

In children of developmental age younger than 4 years, the Mullen Scales of Early Learning subscale Visual Perception was used to assess visual perceptual abilities. Although this test was not a part of the routine examination in CVI (Boonstra et al., 2022), it was included to obtain a measure of visual perception and developmental age equivalents in young children in this study. For children of developmental age 4–11 years, the Developmental Test of Visual Perception-third edition (DTVP-3) was used (Brown, 2016). For children of developmental age above 11 years and older, the DTVP-A^3^ was adopted as a measure of visual perceptual abilities. A neuropsychologist categorized the test results as visual perception deficit (VPD) present or absent.

### Statistical analysis

The extensive test battery contained the following 10 variables: binocular distance visual acuity (log MAR), binocular contrast sensitivity (logCS), ocular alignment (abnormal or normal), eye movement analysis [RTFc (ms), RTFm (ms), and GFA (deg^2^)], VFD (present or absent), small optic disk (yes or no), pale optic disk (yes or no), and VPD (present or absent). The limited test battery contained four variables: binocular visual acuity (log MAR), binocular contrast sensitivity (logCS), ocular alignment (abnormal or normal), and VPD (present or absent).

First, a principal component analysis (PCA) was carried out separately for both test batteries to reduce dimensionality and to make continuous variables, a prerequisite for cluster analysis. Components were included if they had an eigenvalue >1 (Kaiser Criterion). Following this, an unsupervised learning cluster analysis with *k*-means^4^ (Hartigan and Wong, 1979; Amstutz et al., 2022; JothiPrabha et al., 2023) was used to group observations on the included components into clusters sharing common characteristics. Euclidean distance was used as the distance measure in the *k*-means cluster analysis. The analysis was carried out with 25 iterations. Starting with two clusters (*k* = 2), *k* was increased until the optimal number of clusters for each test battery was obtained. The optimal number of clusters was determined primarily by identifying the onset of flattening of the elbow plot of the concerning test battery [an elbow plot presents the total within-cluster sum of squares (WSS) as a function of *k*]. We also explored adjacent *k* values, and compared these to the originally chosen *k* value by evaluating cluster-compactness through visual inspection of the cluster plots. After cluster analysis, we compared functional and structural measures between the clusters (i.e., the CVI subtypes), for both test batteries. For this, we used the Kruskal-Wallis test for continuous measures and the chi-square test for categorical measures. If the clusters differed significantly for a measure, *post hoc* analysis was performed. Dunn’s test was used as a post-hoc test for Kruskal–Wallis results and comparison of standardized residuals with Bonferroni correction for the chi square test. Alpha was set on 0.05. A final clinical expert interpretation was conducted on the chosen cluster solutions to characterize the CVI subtypes. Data were analyzed using R studio version 4.2.0.^5^

## Results

Out of 75 subjects that were identified, data from 51 patients could be used for the main analysis due to missing data in 24 subjects. The main reason for an incomplete visual function assessment was motor and cognitive constraints due to developmental disabilities (*n* = 20) followed by nystagmus and ocular motor restrictions (that affected eye tracking recordings, *n* = 4). An overview of characteristics of patients with a complete visual function assessment is shown in Table 1 (for an overview of patient characteristics and visual function outcomes of patients with an incomplete visual function assessment see Supplementary Tables S2, S3).

**Table 1:** Characteristics of patients with a complete visual function assessment (*n* = 51).

**Demographics**	
Age in years [mean (SD)]	9.7 (2.9)
Gender [*n* (percentage) female]	23 (45)
Gestation length in weeks [mean (SD)]	35.5 (6.4)
Birth weight in grams [mean (SD)]	2,635 (881)
Type of etiology *	
Acquired [*n* (percentage)] Prenatal Perinatal Postnatal	30 (59) 16 (32) 27 (54) 7 (14)
Genetic [*n* (percentage)]	12 (24)
Combined [*n* (percentage)]	7 (14)
Unknown [*n* (percentage)]	2 (4)
Presence of refractive error based on spherical equivalent [*n* (percentage)]	30 (59)
Nystagmus [*n* (percentage)]	7 (14)

^*^: Mutually inclusive (i.e., may co-occur).

Following Boot et al. (2010) and Bosch et al. (2014), the etiologies responsible for CVI were categorized as acquired (*n* = 30), which included prenatal, perinatal, and postnatal causes, genetic (*n* = 12), and combined (i.e., both acquired and genetic simultaneously present; *n* = 7). Prenatal CVI was due to congenital brain anomalies. The predominant perinatal causes included Periventricular Leukomalacia (PVL; *n* = 13) followed by Neonatal Encephalopathy (*n* = 9). Postnatal causes included central nervous system infection (*n* = 4) and post-surgery or post trauma cerebral damage (*n* = 3). See Table 1.

### PCA outcome

**Figure 1:**
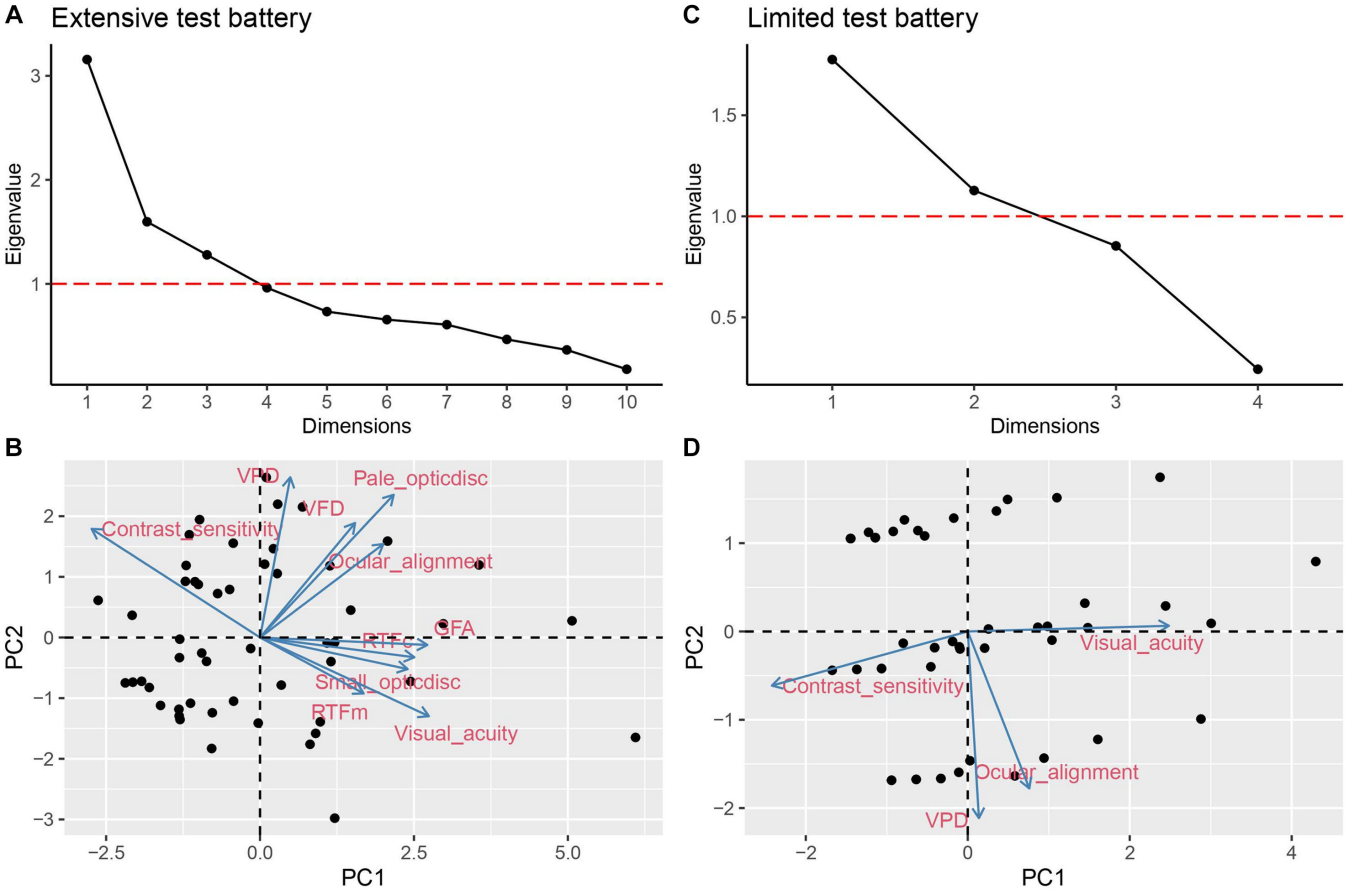
PCA scree plot **(A)** and biplot **(B)** of extensive test battery. Scree plot **(C)** and biplot **(D)** of the limited test battery. Horizontal dashed line in **(A)** and **(C)** indicate eigenvalue = 1.

A PCA for the extensive test battery resulted in three PCs that explained 60% of the total variance. For the limited test battery, there were two PCs that explained 72% of the total variance. Figures 1A,C present the corresponding scree plots. Therefore, three PCs in the extensive battery and the two PCs in the limited battery can represent the extensive test battery (10 variables) and the limited test battery (4 variables) data, respectively. The PC component loadings are displayed in Table 2. Figures 1B,D present the biplots for the first two PCs of the extensive (Figure 1B; bottom left panel) and limited test battery (Figures 1D; bottom right panel).

**Table 2:** Component loadings of the principal components for the extensive and limited test battery.

	**Extensive test battery**			**Limited test battery**	
**Variable**	**PC1**	**PC2**	**PC3**	**PC1**	**PC2**
Visual acuity	0.39	−0.26	0.23	−0.69	−0.02
Contrast sensitivity	−0.39	0.36	−0.36	0.68	−0.21
Ocular alignment	0.28	0.31	−0.38	−0.21	−0.62
VPD	0.07	0.53	0.36	0.03	−0.74
RTFc	0.35	−0.06	0.16		
GFA	0.38	−0.02	−0.25		
RTFm	0.24	−0.18	−0.53		
VFD	0.22	0.38	0.32		
Small optic disk	0.34	−0.10	0.03		
Pale optic disk	0.31	0.47	−0.20		

PC, principal component; RTFc, reaction to fixation cartoon; GFA, gaze fixation area; RTFm, reaction to fixation motion; VPD, visual perception deficit; VFD, visual field defect.

For the extensive test battery, visual acuity, pale optic disk, contrast sensitivity, and VPD contributed the most to the PCA (see Figure 1B). For the first principal component (PC1), factor loadings were highest [i.e., ≥ (−) 0.35] for visual acuity, contrast sensitivity, RTFc, GFA, and small optic disk. For the second principal component (PC2), factor loadings were highest for VFD, pale optic disk, and VPD. For the third principal component (PC3), RTFm and ocular alignment showed the highest contribution. For the limited test battery, evaluation of VPD contributed the most to the PCA (see Figure 1D). Ocular alignment and VPD contributed most to PC1, while visual acuity and contrast sensitivity contributed most to PC2.

### Cluster analysis outcome

**Figure 2:**
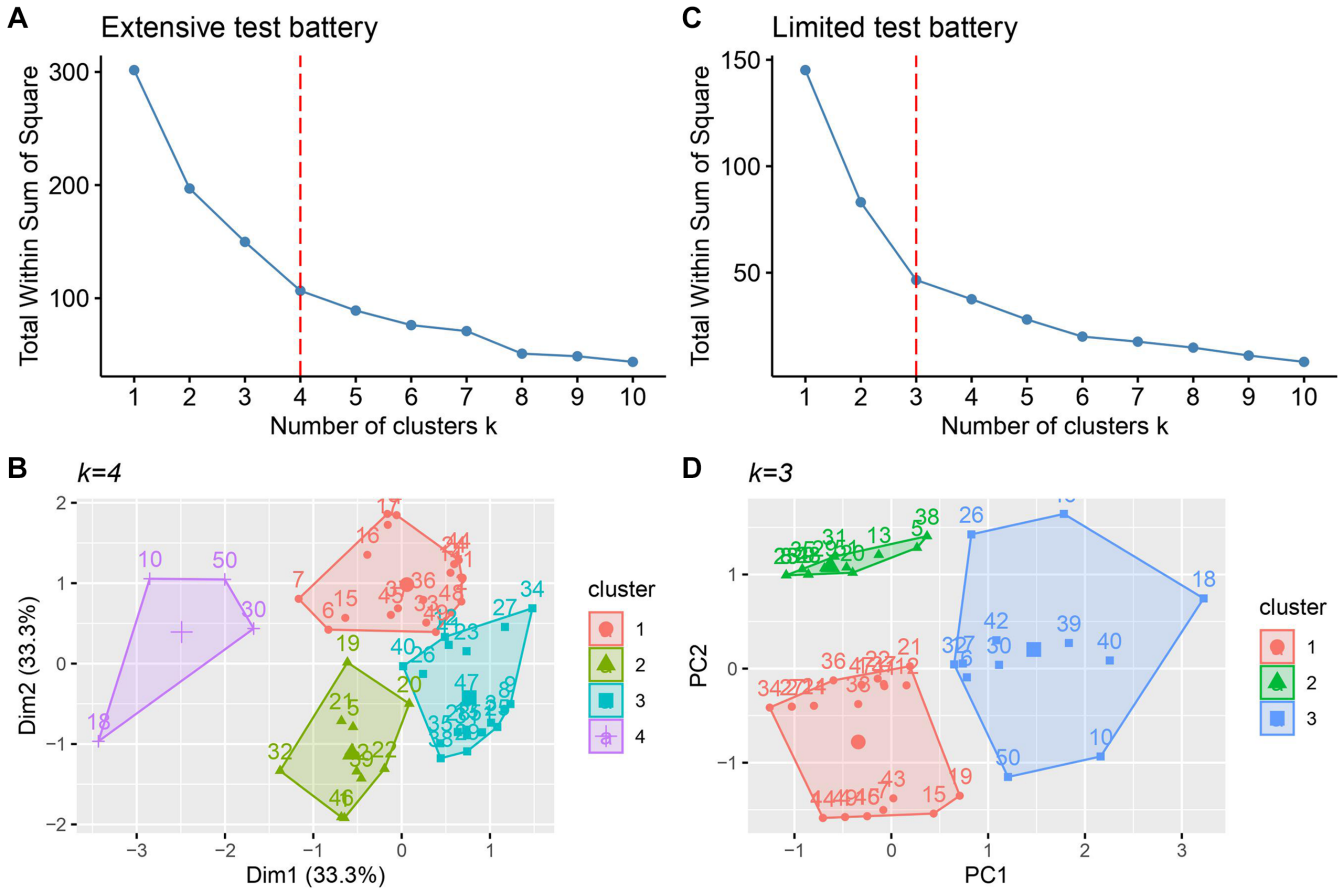
Elbow plot **(A)** and cluster plot **(B)** for the extensive test battery. Elbow plot **(C)** and **(D)** cluster plot **(D)** for the limited test battery. Vertical dashed lines indicate the optimal *k* value.

Using three PCs for the extensive and two PCs for the limited test battery, a *k*-means cluster analysis was performed for both test batteries. Figures 2A,C show the elbow plots for the extensive and limited test battery, respectively. Flattening occurs at *k* = 4 for the extensive test battery and at *k* = 3 for the limited test battery. Figures 2B,D show the corresponding cluster plots. Exploring adjacent *k* values did not result in a more robust or more meaningful clustering, and for that reason we stayed with the originally determined *k* values.

### Comparing and defining the CVI subtypes identified by cluster analysis

For the extensive test battery, there were significant differences between CVI subtypes for nine out of 10 measures (Table 3). For the limited test battery, CVI subtypes differed for all four measures (Table 4). Below, a description is provided for all significant clinical characteristics for the CVI subtypes identified by the extensive and the limited test battery followed by a qualitative expert-based interpretation CVI subtype definition.

**Table 3:** Between group comparison of visual functions for the extensive test battery.

**Measure**	**Subtle characteristics (*n* = 19)**	**Higher-level visual function deficits (*n* = 18)**	**Lower-level visual function deficits (*n* = 10)**	**Higher- and lower- level visual function deficits (*n* = 4)**	**Between group comparison (95% CI)**	**Pairwise comparisons**
Binocular visual acuity (Log MAR) [median (IQR)]	0.10 (0.00–0.20)	0.10 (0.00–0.30)	0.30 (0.20–0.40)	0.55 (0.42–0.75)	*H* = 17.94, *p* < 0.001*, η2 = 0.208	1–3: *Z* = –3.086, *p* = 0.006 1–4: Z = –2.444, *p* = 0.005 2–3: *Z* = –3.326, *p* = 0.021 2–4: *Z* = –2.874, *p* = 0.008
Contrast sensitivity (Log CS) [median (IQR)]	1.80 (1.60–1.90)	1.90 (1.80–1.90)	1.60 (1.27–1.80)	1.15 (0.70–1.30)	H = 16.84, *p* = 0.001*, η2 = 0.159	1–4: *Z* = 2.915, *p* = 0.010 2–3: *Z* = 2.685, *p* = 0.014 2–4: *Z* = 3.623, *p* = 0.001
Deviating ocular alignment [*n* (percentage)]	1 (5)	11 (61)	7 (70)	3 (75)	χ^2^ = 18.07, *p* < 0.001*, *V* = 0.595	Subtype 1 (present - SR: −4.208 vs. absent- SR: 4.208), *p* = 0.002
RTFc (ms) [median (IQR)]	238 (206–251)	245 (206–269)	252 (237–309)	301 (209–390)	*H* = 6.87, *p* = 0.076	n.a.
GFA (deg^2^) [median (IQR)]	2.00 (1.88–2.16)	2.15 (1.76–2.43)	3.12 (2.57–3.51)	4.47 (2.67–4.80)	*H* = 23.51, *p* < 0.001*, η2 = 0.275	1-3: *Z* = –3.687, *p* < 0.001 1–4: *Z* = –3.688, *p* = 0.001 2-3: *Z* = –2.881, *p* < 0.001 2–4: *Z* = –3.120, *p* = 0.003
RTFm (ms) [median (IQR)]	376 (331–438)	414 (343–495)	580 (499–737)	489 (414–609)	*H* = 14.53, *p* = 0.002*, η2 = 0.117	1–3: *Z* = –3.718, *p* = 0.001 2–3: *Z* = –2.626, *p* = 0.025
VFD [*n* (percentage)]	2 (10)	12 (67)	1 (10)	4 (100)	χ^2^ = 22.38, *p* = <0.001* V = 0.662	Subtype 2 (present - SR = 3.208 vs. absent - SR = −3.208) *p* = 0.010
Small optic disk [*n* (percentage)]	1 (5)	1 (6)	2 (20)	3 (75)	χ^2^ = 15.17, *p* = 0.002* V = 0.747	Subtype 4 (present - SR = 3.709 vs. absent- SR = −3.709) *p* = 0.001
Pale optic disk [*n* (percentage)]	0 (0)	14 (78)	4 (40)	4 (100)	χ^2^ = 28.53, *p* = <0.001* V = 0.545	Subtype 1 (present - SR = –4.792 vs. absent- SR = 4.792) *p* < 0.001 Subtype 2 (present - SR = 3.689 vs. SR = –3.689 absent) *p* = 0.001
VPD [*n* (percentage)]	5 (26)	15 (83)	1 (10)	4 (100)	χ^2^ = 19.55, *p* = <0.001* V = 0.619	Subtype 2 (present - SR = 3.833 vs. absent - SR = −3.833) *p* = 0.010

VPD, visual perception deficit; VFD, visual field defect; RTFc, reaction to fixation cartoon (ms); GFA, gaze fixation area (degree); RTFm, reaction to fixation motion (ms); H, Kruskal wallis *H* test; χ^2^, chi square; V, Cramer’s *V*;* Statistically significantly different at *p* < 0.05.

**Table 4:** Between group comparisons of visual functions for the limited test battery.

**Measure**	**Subtle CVI characteristics (*n* = 15)**	**Higher-level visual function deficits (*n* = 24)**	**Higher- and lower- level visual function deficits (*n* = 12)**	**Between group comparison (95% CI)**	**Pairwise comparisons**
Binocular visual acuity (Log MAR) [median (IQR)]	0.10 (0.00–0.20)	0.20 (0.00–0.20)	0.50 (0.40–0.67)	*H* = 25.63, *p* < 0.001* η2 = 0.487	1–3: *Z* = 4.274, *p* < 0.001 2–3: *Z* = 4.715, *p* < 0.001
Contrast sensitivity (LogCS) [median (IQR)]	1.90 (1.60–1.90)	1.90 (1.80–1.90)	1.30 (1.05–1.30)	*H* = 28.58, *p* < 0.001* η2 = 0.552	1–3: *Z* = -4.128, *p* < 0.001 2–3: *Z* = -5.196, *p* < 0.001
Deviating ocular alignment [*n* (percentage)]	0 (0)	16 (67)	6 (50)	χ^2^ = 17.02, *p* < 0.001* V = 0.577	Subtype 2 (present: SR = −4.015 vs. absent: SR = 4.015) *p* < 0.001 Subtype 3 (present: SR = 3.198 vs. absent: SR = −3.198) *p* = 0.008
VPD [*n* (percentage)]	0 (0)	18 (75)	6 (50)	χ^2^ = 20.89 *p* < 0.001* V = 0.640	Subtype 2 (present: SR = –4.346 vs. absent: SR = 4.346) *p* < 0.001 Subtype 3 (present: SR = 3.769 vs. absent: SR = –3.769) *p* < 0.001

VPD, visual perception deficit; H, Kruskal–Wallis *H* test; χ^2^, chi square; a, median (IQR); b, frequency *n* (%); * Statistically significantly different at *P* < 0.05.

#### Extensive test battery

For details see Table 3.

Subtype 1 showed an overall relatively higher performance on all measures compared to other subtypes. Therefore, we characterized this subtype as “subtle characteristics” CVI.

Subtype 2 displayed a higher number of VPDs combined with near normal visual acuity. This was accompanied with VFDs and pale optic disks. So, we termed this subtype as “higher-level visual function deficits” CVI.

Subtype 3 showed reduced lower-order functions such as visual acuity and contrast sensitivity combined with low occurrence of higher-level defects with abnormal eye movement behavior. Hence, this subtype was called “lower-level visual function deficits” CVI.

Subtype 4 had both the higher- and the lower-level visual functions affected, along with ocular structural defects. Therefore, we defined this as “higher- and lower- level visual deficits” CVI.

#### Limited test battery

For details see Table 4.

Subtype 1 showed normal to near normal characteristics and for that reason, we termed this subgroup as “subtle characteristics” CVI.

Subtype 2 displayed VPDs combined with near normal visual acuity. For that reason, we termed this subgroup as “higher-level visual function deficits” CVI.

Subtype 3 consisted of children in which both the higher- and the lower-level visual functions were affected. Therefore, we defined this subgroup as “higher- and lower- level visual deficits” CVI.

### Comparison of patient classification between the two test batteries

Table 5 shows the classification of the 51 patients according to both test batteries. As can be seen in this table, most patients were classified similarly for the three subtypes that were present in both subtyping systems (subtle characteristics, higher-level visual function deficits, and higher- and lower- level visual function deficits); in only two patients, there was a clear deviation (‘higher- and lower- level visual function deficits’ according to the limited test battery and ‘subtle characteristics’ according to the extensive test battery; see Discussion section). Patients who were attributed to the subtype that is only present in the extensive test battery (lower-level visual function deficits) could be attributed to any subtype when using the limited test battery (Table 5, last column).

**Table 5:** Classification outcomes of the extensive and limited test battery (*n* = 51).

	Extensive test battery				
Limited test battery		Subtle characteristics	Higher-level visual function deficits	Higher- and lower- level visual function deficits	Lower-level visual function deficits
	Subtle characteristics	**12**	1	0	2
	Higher-level visual function deficits	5	**15**	0	4
	Higher- and lower- level visual function deficits	2	2	**4**	4

Extensive test battery (vertical) and Limited test battery (horizontal). Bold values indicate the CVI patients similarly classified with both test batteries.

### Patient characteristics of CVI subtypes of the extensive test battery

**Figure 3:**
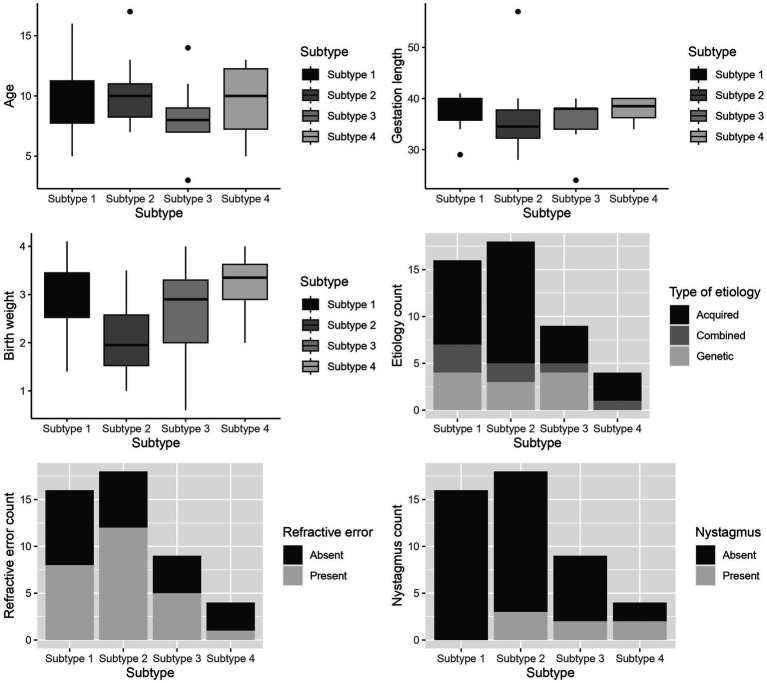
Box and whiskers plot of the patient characteristics between the subtypes of the extensive test battery for - age (years), gestation length (weeks), birth weight (grams), Etiology, Refractive error, and Nystagmus. Subtype 1- Subtle CVI characteristics, subtype 2- higher-level visual function deficits, subtype 3- lower-level visual function deficits, subtype 4: higher- and lower-level visual function deficits.

A between-subtype analysis of patient characteristics (age, gestation length, birth weight, etiology, refractive error, and nystagmus) of the extensive test battery is shown in Figure 3. This revealed significant differences between the subtypes of the extensive test battery in gestation length (*H* = 8.452, *p* = 0.037), birth weight (*H* = 8.769, *p* = 0.032), and presence of nystagmus (χ^2^ = 7.93, *p* = 0.047). In the *post hoc* analysis, patients of the higher-level visual function deficits subtype had a statistically lower gestation length compared to the subtle characteristics subtype (median [IQR] 34 [32–38] versus 39 [36–40] weeks; *p* = 0.005) and a lower birth weight compared to patients of the subtle characteristics subtypes (1800 [1375–2,625] versus 2,900 [2300–3,300] grams; *p* = 0.005) and higher- and lower- level visual function deficits CVI subtype (3,350 [2300–3,875] grams; *p* = 0.012). Presence of nystagmus was not statistically different between the groups.

## Discussion

The first aim of this study was to classify CVI as subtypes with an extensive test battery and a limited test battery in a subset of CVI patients with mild to moderate visual impairment. The second aim was to compare the outcome of a data-driven subtyping method of an extensive CVI test battery with that of a limited test battery. This resulted in four CVI subtypes for the extensive test battery, which were named subtle characteristics group, higher-level visual function deficits group, lower-level visual function deficits group, and higher- and lower- level visual function deficits group. With the limited test battery, three CVI subtypes were identified, which were named subtle characteristics group, higher-level visual function deficits group, higher- and lower- level visual function deficits group. For the three subtypes present in both batteries, the majority of the patients were classified similarly by both subtyping systems; patients attributed to the fourth subtype (i.e., lower-level visual function deficits CVI) in the extensive test battery were attributed to different subtypes when using the limited test battery. Patients belonging to the higher-level visual function deficits subtype had a lower birth weight and gestation length than the other patients. These were patients with perinatal damage.

In the past decades, CVI has been considered as a general diagnosis (Boot et al., 2010). Recently, Sakki et al. (2021) took the first steps towards classifying CVI. The limited test battery in the current study resembled the five basic-vision related measures (i.e., visual acuity, contrast sensitivity, stereopsis, visual perception) used in Sakki et al. (2021) to acquire their classification. Although there were discrepancies in the types of tests used for the measurements, the attempt to replicate a similar test battery as Sakki et al. was successful based of the similarities in the subtyping between both study outcomes. For example, with the limited test battery, the subtle CVI subtype group was like group A1 and the higher-level visual function deficits subtype group was like group A2 in Sakki et al.; discussed further below. On the other hand, our study differs from Sakki et al. based on two approaches. We included additional input variables critical for CVI diagnosis, as described in a recent CVI diagnostic guideline, such as eye tracking behavior, VFDs, and optic disk abnormalities (Boonstra et al., 2022). This made up the extensive test battery. Secondly, Sakki et al. reported a ‘severity-based’ subtype classification through hierarchical agglomerative cluster analysis. Whereas in our study, a ‘characteristics-based’ subtype classification was used with the help of *k*-means cluster analysis. A severity-based classification according to Sakki et al. resulted in a broad-range subtyping whereas a characteristics-based subtype classification as the current study has broken down the clinical presentation of CVI into subtypes where role of every visual function and structural measure is evident. The latter is arguably more valuable in clinical practice for targeted CVI diagnosis according to Boot et al. (2010).

A PCA with the limited test battery identified two distinctive components: (1) a component loading high on basic visual function measures (PC1: visual acuity and contrast sensitivity) and (2) a component loading on higher-order and ocular alignment variables (PC2: VPD and ocular alignment). The identification of these two components is in line with the components found by Sakki et al. (2021). This also confirms our attempt to use similar measures that have been previously used in a data-driven classification such as Sakki et al. (2021). With the extensive test battery three components were found: (1) a component loading high on basic visual function and fixation measures (PC1: visual acuity, contrast sensitivity, small optic disk, RTFc and GFA), (2) a component loading high on visual pathway measures and higher-level visual function deficits (PC2: VFDs, pale optic disk and VPDs), and (3) a component showing high factor loadings on the variables motion processing and ocular alignment (PC3). These results indicate that the components identified by the PCA using an extensive test battery (including visual pathway and eye movement measures) compared to a limited test battery show more distinctive and fine-grained clusters of visual deficits. It is remarkable that GFA loads in the PC1 because one could argue that stable fixation is related to alignment and motion. However, an enlarged GFA is related to the development of fixation and could be a sign of CVI and is often related to lower visual acuity and more crowding (Kooiker et al., 2016). The *k*-means cluster analysis resulted in the identification of four CVI subtypes for the extensive test battery and three CVI subtypes for the limited test battery. The patient classification outcomes of both test batteries of our study showed overlap for the subtle characteristics, the higher-level visual function deficits, and the higher- and lower- level visual function deficits subtypes (Table 5). On the other hand, there were differences in classification outcomes between the extensive and limited test battery, which is discussed below for every subtype to evaluate the added value of an extensive test battery.

The ‘subtle CVI characteristics’ subtype group showed near normal visual acuity with better outcomes on all measures compared to other groups. This agrees with a previous study that identified CVI in children with an abnormal medical history but good visual acuity (Van Genderen et al., 2012). The subtle CVI subtype is similar to Group A1 in Sakki et al. (2021) where near normal visual acuity, contrast sensitivity was seen in the presence of normal visual perception evaluation as compared to healthy children. Both test batteries resulted in a subtle CVI subtype, and in most cases, the test batteries agreed on classifying patients as belonging to this subtype (Table 5). On looking into differences in classification outcomes between the two test batteries, patients who were classified as subtle CVI subtype with the extensive test battery, were classified as belonging to either higher-level or higher- and lower- level visual function deficits subtypes with the limited test battery (Table 5). This was due to the presence of VFDs, pale optic disks along with larger GFAs; thereby not qualifying them as having subtle CVI characteristics. Two patients showed an apparently larger classification deviation (i.e., belonging to the subtle group in the extensive test battery and to the higher- and lower- level visual function deficits subtype in the limited test battery). This was because these patients had a low visual acuity and contrast sensitivity with deviating ocular alignment, but their visual pathways test and eye-movement behaviors were normal compared to other groups; hence classifying them as subtle with the extensive test battery. In general, low visual acuity and low contrast sensitivity are signs of severe CVI. Since the limited test battery was based on only core vision measures, in our case, the added measures (extensive test battery) have created differences in the classification outcomes. The subtypes obtained from the limited test battery resemble the features of group A1 in Sakki et al. (2021). With the extensive test battery, we found that patients of subtle subtype also had a slight delay in cartoon RTFs according to Kooiker et al. (2014) who classified RTFc of children with visual processing impairments as ‘fast,’ ‘medium’ or ‘severe.’ This subtype of children fell into the ‘fast risk group’ of cartoon RTF (range: 171–240 ms). Delayed responses to a highly salient stimulus such as cartoon (combination of contrast, motion, and form) prompts the involvement of extrastriate pathways in the brain (Kooiker et al., 2014). RTFc is a “rough” measure that may discriminate between a developed ocular motor system and an underdeveloped or “young” oculomotor system, without being specific for CVI. However, children belonging to this subtype, with only a limited test battery, may have the risk of being misdiagnosed as ‘normal’. Therefore, using an extensive test battery becomes pivotal in diagnosing this subtype of CVI and differentiating children with CVI from healthy children.

The ‘higher-level visual function deficits’ subtype is a subtype with near normal visual acuity, higher number of VPDs, VFDs, and pale optic disks. This subtype in our study is in agreement with group A2 reported by Sakki et al. (2021) where near normal visual acuity and contrast sensitivity with reduced visual perception scores compared to group A1 were found. However, unlike Sakki et al. (2021) our study included variables containing information about visual field and optic disk abnormalities. Patients classified as belonging to this subtype with the extensive test battery were classified mainly (15 of 18) as higher-level visual function deficits by the limited test battery as well. Some (3 of 18) were classified as subtle or higher- and lower- level visual function deficits CVI with the limited test battery due to absence or presence of VPD, visual field and pale optic disk, and reduced visual acuity (Table 5). Broadening of the test battery has identified whether a patient belongs in this subtype. The subtyping differs based on, firstly, the presence or absence of VPDs in the presence of subnormal acuity in this group (i.e. limited test battery features) similar to group A2 in Sakki et al. VPDs are found commonly in CVI (Chandna et al., 2021) and are primarily due to the involvement of the extrastriate regions in the brain such as the ventral and dorsal streams (Macintyre-Béon et al., 2013). Studies have shown that visual functions can improve in children with CVI (when cortically impaired) due to plasticity (Kozeis, 2010). This could possibly be the reason for normal visual acuity in this subtype with damages in higher visual centers. Secondly, the classification outcomes are affected when there is presence of VFDs and optic disk pallor. In CVI, optic atrophy is due to ischemia or compression that occur independently or through a secondary neuronal loss of the retinal layers and optic nerve (Ruberto et al., 2006; Jacobson et al., 2019). This is also in agreement with Sakki et al. (2021) where they report several VFDs (not included in the classification) in group A2. In addition, this subtype has a higher number of perinatally acquired etiologies with lower gestation length (Figure 3) due to PVL and neonatal asphyxia which could have caused the VPDs and the visual pathway damages. This agrees with Bosch et al. (2014) where they also found a higher number of VPDs and visual field defects and also different visual field defects in children with acquired etiology compared to genetic etiology. This could also have driven the classification outcomes. However, future studies including etiology in CVI subtyping might aid in better understanding.

The ‘higher- and lower- level visual function deficits CVI’ subtype group showed reduced visual acuity with overall deficits in all the outcome measures. Sakki et al. (2021) included participants with moderate to low visual acuity and contrast sensitivity in the same range as our higher- and lower- level visual function deficits CVI group; however, such a subtype was not evident in their study due to methodological differences between both studies. Patients who were classified as higher- and lower- level visual function deficits subtype with the extensive test battery were all classified as belonging to the same subtype with the limited test battery as well (Table 5). Some patients were classified as higher- and lower- level visual function deficits subtype with the limited test battery but as subtle or lower-level or higher-level subtypes with the extensive test battery (Table 5). This was due to absence of VPDs, small optic disk, and larger GFAs and delayed RTFm. The classification with the extensive test battery is based on involvement of all areas of vision processing such as: lower order, higher order, visual pathway and visual orienting responses (Fazzi et al., 2007; Macintyre-Béon et al., 2013; Kooiker et al., 2016; Jacobson et al., 2020). Previous literature shows the co-occurring involvement of these areas in CVI. Kooiker et al. (2016) reported a correlation between GFA and RTFm with visual acuity and perception dysfunction (i.e., VPD). Optic disk damages are due to incomplete retinal ganglion cell formation in early visual development thereby associated with poor visual acuity; when the optic nerve is atrophic and not hypoplastic, this usually indicates injury to the retinal ganglion cells (Hoyt, 2003; Fieß et al., 2018). These ONH damages have proved to cause visual fields defects. In contrast, the classification with only core vision measures with the limited test battery excluded CVI patients with deficits in visual pathway or visual orientation and therefore probably belonged in other subtypes.

The ‘lower-level visual function deficits’ subtype group was identified only with the extensive test battery. This subtype consisted of patients from all the subtypes of the limited test battery: subtle, higher-level, and higher- and lower- level visual function deficits CVI (Table 5). This subtype was not seen in the subgrouping by Sakki et al. (2021) because this subtype was evident only due to the use of eye tracking behavior and visual pathway tests.

In this subtype we see reduced lower order visual defects with minimal or no VPDs probably as a result of retrograde degeneration of ganglion cells. In a follow up study Sakki et al. (2022) compared their severity-based classification with MRI brain lesions and found no associations between visual function score and MRI lesions. However, another study showed a correlation between visual function score (without visual perception) and subcortical structures, corpus callosum, and cerebellum (Tinelli et al., 2020). This means that lesions in these areas could have caused reduced lower-level visual function in this subtype. In addition, there were larger GFAs and delayed motion RTFs compared to other subtypes. There can be several reasons for this. This subtype had patients with the lowest age (Figure 3). and age might influenced eye-tracking outcomes in CVI (Hadad et al., 2015; Kooiker et al., 2015); eye tracking data were compared to a large subset of typically developing children in whom age also influences eye tracking outcomes. Studies show that abnormal visual orienting responses are attributed to reduced visual functioning. This is because the temporal and spatial information from the visual cortex is damaged or degraded to execute visually guided behaviors such as eye movements (Kelly et al., 2021). Therefore cerebral abnormalities could have caused a higher percentage of deviating ocular alignment, larger GFAs, and longer RTFm times in addition to lower-order visual functions in this group (Tinelli et al., 2020). Also, this subtype had a relatively higher number of genetic etiologies compared to other groups. This is in agreement with Bosch et al. (2014) in which they showed a higher prevalence of reduced visual functioning, strabismus, visual field and ONH defects in children with CVI due to genetic etiologies compared to children with CVI due to acquired etiologies. However, in this study, there were only 10 patients in the lower-level visual deficits group and more insight about this subtype of CVI warrants a larger sample size.

In addition to this, Sakki et al. (2021) reported patients (group B in their study) with an incomplete test battery mainly due to the presence of developmental disabilities who could not be included in the classification analysis. This reconfirms that children with CVI often present with multiple disabilities and any attempt to classify CVI in the future, might result in a ‘low functioning range’ subtype.

To summarize, the subtle characteristics, higher-level visual function deficits and the higher- and lower- level visual function deficits subtypes occurred in both test batteries; however, the extensive test battery brought out additional core features of these subtypes and led to uncovering the lower-level visual function deficits subtype. This is one of the first few studies to exhibit a characteristic based subtype classification of childhood CVI. We believe that this subtyping finally can aid in simplifying the complexity of CVI and give a better specification of subtypes. We consider this as the main strength of our study. We acknowledge the limitations of this study. Participants underwent different kinds of tests for visual acuity, contrast sensitivity, visual fields, and visual perception based on their development which was further used in the analysis. This attempt to include different assessment methods could have influenced the outcomes of the classification. Developmental age could not be included as one of the study variables due to lack of information in the medical records. Future prospective studies should include developmental age as an external measure. Due to discrepancies in the retrospective medical records, we used visual perception, visual fields and optic disk measures as categorical variables graded by professionals. This may have caused subjective bias and influenced the PCA contribution (Figure 1B). In this study we included only small optic disk (i.e., ONH hypoplasia) as a variable in the cluster analysis but we recommend that in the future, optic nerve and visual fields should be replaced with global measures of ONH from optical coherence tomography (OCT) and perimetry. Since this was the first time that a ‘characteristics-based’ subtyping was established, we included all necessary measures in the extensive test battery. Our study outcomes show that every measure in the extensive test battery has contributed in defining a subtype. Also, due to the heterogeneity of CVI it is not easy to develop tailor made interventions as different CVI-types seem to need a different approach. However, we foresee that the CVI classification established in this study can be useful when a prognosis or intervention is needed. This study is an attempt to establish an inventory to make recognizable CVI groups that can be helped with suitable advices and interventions. Further, we strongly feel the need to (i) find a common ground in the number of investigations used between an extensive (*n* = 10) and a limited test battery (*n* = 4), which might be achievable by combining the outcomes of some of the measures, and (ii) test the obtained CVI classification in the clinical population. These steps can eventually aid in effective diagnosis in pediatric CVI with a small evidence based subset of tests that can lead to strategic intervention and prognosis.

## Conclusion

This study has compared CVI subtype classifications found with an extensive compared to a limited test battery. Our findings indicate that it is beneficial to use an extensive test battery over a limited test battery to provide a finely meshed set of assessments for CVI diagnosis which could lead to more targeted interventions and rehabilitation. Moreover, our study highlights the value of including visual fields, optic disk changes and eye movement analysis with eye tracker in a CVI subtype classification. These make a substantial difference in the clarity with which CVI subtypes can be identified.

## Data availability statement

The original contributions presented in the study are included in the article/Supplementary material, further inquiries can be directed to the corresponding author.

## Ethics statement

For the use of data collected during regular clinical care an exemption from ethical review was obtained from the METC of Oost-Nederland (MEC 2021–13169). The studies were conducted in accordance with the local legislation and institutional requirements. Written informed consent for participation in this study was provided by the participants’ legal guardians/next of kin.

## Author contributions

JP: Data curation, Formal analysis, Writing – original draft. BH: Methodology, Supervision, Writing – review & editing. NJ: Conceptualization, Supervision, Writing – review & editing. AC: Writing – review & editing. FB: Conceptualization, Methodology, Supervision, Writing – review & editing.

## Footnotes

^1^Clinical software in optometry a (opticaldiagnostics.com)

^2^LEA SYMBOLS® Near Vision Card – Good-Lite

^3^(DTVP-A) Developmental Test of Visual Perception–Adolescent and Adult (wpspublish.com)

^4^kmeans function - RDocumentation

^5^http://www.rstudio.com
