# Corrigendum: Childhood cerebral visual impairment subtype classification based on an extensive versus a limited test battery

**DOI:** 10.3389/fnins.2024.1462687

**Published:** 2024-08-20

**Authors:** Jannet Philip, Bianca Huurneman, Nomdo M. Jansonius, Antonius H. N. Cillessen, Frouke N. Boonstra

**Affiliations:** ^1^Royal Dutch Visio, National Foundation for the Visually Impaired and Blind, Huizen, Netherlands; ^2^Behavioural Science Institute, Radboud University, Nijmegen, Netherlands; ^3^Department of Cognitive Neuroscience, Donders Institute for Brain, Cognition and Behaviour, Radboud University Medical Centre, Nijmegen, Netherlands; ^4^Department of Ophthalmology, University Medical Center Groningen, University of Groningen, Groningen, Netherlands; ^5^Graduate School of Medical Science, University Medical Center Groningen, University of Groningen, Groningen, Netherlands

**Keywords:** cerebral visual impairment, childhood, subtyping, classification, visual function, eye movements, visual fields, optic disk

In the published article, there was an error. The following text should be removed: “For the eye tracking data, the study was approved by the Medical Ethical Research Committee (METC) of the Erasmus Medical Center, Rotterdam the Netherlands (MEC 2012–097).” The reason for this removal is that the data used in the published study did not include the data collected under this approval but only the data collected according to the following sentence: “For the use of data collected during regular clinical care an exemption from ethical review was obtained from the METC of Oost-Nederland (MEC 202113169).”

A correction has been made to **Materials and methods**, “*Participants*,” 2nd paragraph. The sentences previously stated:

“For the eye tracking data, the study was approved by the Medical Ethical Research Committee (METC) of the Erasmus Medical Center, Rotterdam the Netherlands (MEC 2012–097). For the use of data collected during regular clinical care an exemption from ethical review was obtained from the METC of Oost-Nederland (MEC 2021–13169). Informed consent was obtained from each patient's parent or caregiver. We adhered to the tenets of the Declaration of Helsinki (2013) for research involving human subjects.”

The corrected sentences appear below:

“For the use of data collected during regular clinical care an exemption from ethical review was obtained from the METC of Oost-Nederland (MEC 2021–13169). Informed consent was obtained from each patient's parent or caregiver. We adhered to the tenets of the Declaration of Helsinki (2013) for research involving human subjects.”

A correction has also been made to the Ethics statement to remove the same text. The correct Ethics statement appears below:

