# Supplementary material for: Childhood cerebral visual impairment subtype classification based on an extensive versus a limited test battery
Source: Front Neurosci. 2023 Oct 26;17:1266201. doi: 10.3389/fnins.2023.1266201 (PMC10637406; doi:10.3389/fnins.2023.1266201)
Supplement: Supplementary file 1 [file Table_1.DOCX]

Supplementary material (Information on patients with an incomplete visual function assessment)

**Table 1:** Overview of functional vision assessment completeness for all patients (N=75)

| **Functional vision measure** | **Developmental age categories (years)** | **Type of test** | **N(%) with a complete assessment(n=51)** | **N(%) with an incomplete assessment (n=24)** |
| --- | --- | --- | --- | --- |
| **Measures used in both limited and extensive CVI test battery** | | | |  |
| Visual acuity  (logMAR) (n(percentage)) | <2 years (preverbal) | TAC | - | 7(29) |
|  | 2-4 (preverbal) | Cardiff | 7(13) | 8(33) |
|  | 3-6 (verbal) | Lea symbols | 9(18) | 6(25) |
|  | >6 | Number chart | 35(68) | 3(12) |
| Contrast sensitivity  (logCS) (n(percentage)) | <2 (preverbal) | Hiding Heidi low contrast face test | - | 14(58) |
|  | 3-5 (preverbal) | Cardiff contrast | 12(23) | 6(25) |
|  | 6 and above | Lea low contrast test | 32(63) | 4(16) |
|  | 6 and above | GECKO | 7(14) | - |
| Ocular alignment (n(percentage)) | 1-18 | Cover/uncover | 51(100) | 24(100) |
| Evaluation of visual perception  (n(percentage)) | <4 | Mullen scale | 10(20) | 8(33) |
|  | 4-11 | DTVP-2/DTVP-3 | 30(59) | 4(16) |
|  | >11 | DTVP-A | 11(21) | - |
| **Measures used only in the extensive CVI test battery** | | | |  |
| RTFc (ms) (n(percentage)) | 1-18 | Tobii T60XL | 51(100) | 14(58) |
| GFA (deg^2^) (n(percentage) | 1-18 | Tobii T60XL | 51(100) | 14(58) |
| RTFm (ms) (n(percentage)) | 1-18 | Tobii T60XL | 51(100) | 9(37) |
| Visual fields*  (n(percentage)) | <5 (preverbal) | Arc perimetry | 17(33) | 22(92) |
|  | > 5(verbal) | Kinetic perimetry | 18(35) | 2(8) |
|  | > 5(verbal +subjective response) | Static perimetry | 16(31) | - |
| Optic disc evaluation (n(frequency)) | 1-18 | Slit lamp (90D) or IDO (20D) | 51(100) | 24(100) |

*Table 1: * mutually inclusive (i.e, can co-occur)*

**Table 2:** Characteristics of patient with incomplete visual function assessment (N=24)

| **Measure** | **Outcome** |
| --- | --- |
|  |  |
| **Demographics** | |
| Age in years (mean(SD)) | 7.3(3.8) |
| Gender (n(percentage)) | Male: 12(50)  Female:12(50) |
| Gestation length in weeks (mean(SD)) | 38.13(3.64) |
| Birth weight in grams (mean(SD)) | 3184(627) |
| **Type of etiology *** (n(percentage)) | |
| Acquired  Prenatal  Perinatal  Postnatal | 12(50)  4(33)  5(42)  3(25) |
|  |  |
| Genetic | 10(41) |
| Combined | 1(4) |
| Unknown | 1(4) |
| Presence of refractive error based on SE (n(percentage)) | 11(46) |
| Nystagmus n(percentage)) | 6(25) |

*Table 2: SE: spherical equivalent, *: Mutually inclusive (i.e. may co-occur)*

**Table 3:** Visual function outcomes of the incomplete group (N=24)

| **Measure** | **Outcome** |
| --- | --- |
| Visual acuity(logMAR) (median(IQR)) | 0.50(0.30-0.60) |
| Contrast sensitivity (logCS) (median(IQR)) | 1.30(1.00-1.50) |
| Deviating ocular alignment (n(percentage)) | 11(46) |
| RTFc (ms) (median(IQR)) | 306(271-379) |
| GFA (deg^2^) (median(IQR)) | 2.55(2.40-2.94) |
| RTFm (ms) (median(IQR)) | 581(443-759) |
| VFD (n(percentage)) | 7(29%) |
| Small optic disc (n(percentage)) | 6(25%) |
| Pale optic disc (n(percentage)) | 10(42%) |
| VPD (n(percentage)) | 4(33%) |

*Table 3: RTFc: reaction to fixation cartoon (ms), GFA: gaze fixation area (degree), RTFm: reaction to fixation motion (ms), VPD: visual perception deficit,*
